# Suppression of Type I Interferon Signaling by *Flavivirus* NS5

**DOI:** 10.3390/v10120712

**Published:** 2018-12-14

**Authors:** Stephanie Thurmond, Boxiao Wang, Jikui Song, Rong Hai

**Affiliations:** 1 Department of Microbiology and Plant Pathology, University of California, Riverside, Riverside, CA 92521, USA; sthur002@ucr.edu; 2 Graduate Program in Cell, Molecular and Developmental Biology, University of California, Riverside, Riverside, CA 92521, USA; 3 Department of Biochemistry, University of California, Riverside, Riverside, CA 92521, USA; bwang030@ucr.edu

**Keywords:** *flavivirus*, ZIKV, NS5, type I IFN antagonist

## Abstract

Type I interferon (IFN-I) is the first line of mammalian host defense against viral infection. To counteract this, the flaviviruses, like other viruses, have encoded a variety of antagonists, and use a multi-layered molecular defense strategy to establish their infections. Among the most potent antagonists is non-structural protein 5 (NS5), which has been shown for all disease-causing flaviviruses to target different steps and players of the type I IFN signaling pathway. Here, we summarize the type I IFN antagonist mechanisms used by flaviviruses with a focus on the role of NS5 in regulating one key regulator of type I IFN, signal transducer and activator of transcription 2 (STAT2).

## 1. Introduction

Flaviviruses are globally significant arthropod-borne viruses that cause disease in hundreds of millions of people each year. The *Flavivirus* genus is part of the *Flaviviridae* family and comprises over 70 species, including dengue, Zika, yellow fever, West Nile, Japanese encephalitis, and tick-borne encephalitis viruses. Facilitated by the warming climate, urbanization, and increasing travel to endemic areas, many of these pathogens have expanded into new territories, and flaviviral infections have increased worldwide [1]. Despite the enormous burden on public health posed by flaviviruses, there are currently no antiviral therapies available and limited vaccines.

To establish successful infection in vertebrates, flaviviruses must first overcome the host antiviral response, which is primarily mediated by type I interferons (IFN-I) [2]. IFN-I stimulates expression of hundreds of genes that disrupt various stages of the viral life cycle. The power and importance of this host defense is demonstrated by the fact that all flaviviruses have evolved elaborate mechanisms to antagonize and evade the type I IFN response. The ability of individual viruses to suppress this pathway determines host and tissue tropisms and severity of disease [3,4]. Therefore, understanding the interaction between the host immune response and viral antagonism of this defense at the molecular level elucidates mechanisms of pathogenesis and facilitates the development of safe and effective antiviral therapies and vaccines.

Flaviviruses employ diverse strategies to subvert the host immune system. To avoid being recognized as “non-self,” which triggers IFN-I production, flaviviruses mask their genome with RNA caps that mimic that of the host and the intermediate RNAs are hidden from cytoplasmic sensors by membranes hijacked by viral replication proteins [5]. Flaviviruses also actively antagonize proteins that function within the IFN-I signaling pathway by inhibiting their post-translational modifications, competing for protein–protein interactions, or targeting them for degradation [2]. These diverse mechanisms of IFN antagonism are typically carried out by flavivirus non-structural proteins.

Non-structural protein 5 (NS5) is the largest and most conserved flavivirus protein [6]. It is responsible for replicating and capping the viral genome, but is also a potent innate immune antagonist in all flaviviruses studied thus far [7]. However, the mechanisms utilized by the NS5 proteins from related viruses have been shown to diverge significantly. This review discusses the IFN-I-mediated defense mounted by flavivirus-infected hosts and the various mechanisms employed by flaviviruses to counteract this defense, with special emphasis given to NS5-mediated suppression of human signal transducer and activator of transcription 2 (hSTAT2)-dependent IFN signaling. Three of the major disease-causing flaviviruses’ NS5 proteins interact with and inhibit hSTAT2, a central regulator of the type I IFN response. We discuss the ways in which this interaction diverges among the highly related flaviviruses and the impact of hSTAT2 inhibition on viral pathogenesis.

## 2. *Flavivirus* Disease and Transmission

Disease caused by flaviviruses ranges from mild symptoms such as fever, rash, and joint pain to more severe illness that includes hemorrhagic fever, encephalitis, and neurological sequelae. However, very few infected individuals experience serious illness, and most are entirely asymptomatic. It is currently not possible to predict an individual’s disease outcome due to our inadequate understanding of the molecular basis of the pathogenesis of severe disease. There are no vaccines available for dengue, Zika, or West Nile viruses. Even for flaviviruses with effective vaccines, such as yellow fever, Japanese encephalitis, and tick-borne encephalitis viruses, outbreaks still occur from time to time in developing countries.

Dengue virus (DENV) is responsible for the highest incidence of disease among the flaviviruses (≈400 million annually) [8]. There are four genetically distinct serotypes of DENV and infection with one serotype confers long-lasting immunity against only the infecting serotype. Primary infections are frequently asymptomatic but can result in fever and rash, whereas secondary infections, especially with a heterologous serotype, can cause dengue hemorrhagic fever and shock syndrome, likely due to antibody-dependent enhancement (ADE) [9]. Zika virus (ZIKV), which emerged recently in several major epidemics in Asia and the Americas, causes similar symptoms to dengue fever and has additionally been causally linked to congenital microcephaly and Guillain-Barré syndrome [10]. West Nile virus (WNV) is common in Africa, the Middle East, and Europe, and appeared in North America in 1999 [11]. In addition to febrile illness, WNV is a major cause of viral encephalitis [12].

The human pathogen flaviviruses are vector-borne, although different tick and mosquito species are utilized by different viruses. Yellow fever virus (YFV), DENV, and ZIKV are transmitted primarily by *Aedes* mosquitoes [13], whereas WNV and Japanese encephalitis virus (JEV) are transmitted through the *Culex* species [14,15]. Flaviviruses are also zoonotic and rely on non-human animal vectors for widespread circulation. For example, pigs and birds are amplifying hosts for JEV [16], and non-human primates are ZIKV amplification hosts [17]. Flaviviruses alternate between two distinct transmission cycles: sylvatic and urban. The sylvatic transmission cycle refers to the transmission of the virus between the arboreal vector and non-human animal hosts, and the urban cycle consists of circulation between arthropods and humans [18]. The recent ZIKV epidemics have revealed additional mechanisms of transmission that may be unique to Zika, such as sexual contact and perinatal transmission [19,20].

Because of the necessity of alternating between vertebrate and arthropod hosts, flaviviruses have adapted their immune restriction mechanisms for distinct species. For example, DENV proteins can cleave the human immune factors stimulator of interferon genes (STING) and STAT2 (discussed below) but not non-human primate STING or murine STAT2 [3,21,22]. Similarly, ZIKV causes the degradation of human but not murine STAT2 [3]. These species-specific immune suppression mechanisms help to explain in part why DENV fails to reach high titers non-human primate models [23,24], and why immunocompetent mice are poor disease models for many flaviviruses, including ZIKV and DENV [25,26].

## 3. *Flavivirus* Genome and Life Cycle

*Flavivirus* genomes are 10-11 kb single-stranded positive-sense RNA molecules flanked by structured 5’ and 3’ UTRs. A recent study demonstrated pervasive higher-order structures throughout the ZIKV [27] and DENV2 RNA genomes, spanning at least eight distinct regions [28]. A virally encoded methyltransferase provides a ^m7^GpppN cap structure to the 5’ end of the genome similar to mammalian mRNA caps [29], but the flaviviral genome lacks a 3’ polyadenylation tail. The genome is translated as a single polyprotein, which is then proteolytically processed by host and viral proteases to generate three structural proteins (capsid (C), pre-membrane (prM), and envelope (E)), and seven non-structural proteins (NS1, NS2A, NS2B, NS3, NS4A, NS4B, and NS5). The non-structural (NS) proteins are responsible for replication of the viral genome, polyprotein processing, and host immune response antagonism. Mature virions are ≈50 μm in diameter and consist of the RNA genome encapsulated by a lipid envelope and the three structural proteins C, prM, and E [6].

Flaviviruses enter the cell via endocytosis and traffic to endosomes where the envelope protein undergoes a low pH-induced conformational change to induce fusion of the endosomal membrane with the viral membrane. This fusion event allows the nucleocapsid to be released from the endosome, and the genome is rapidly translated at the surface of the endoplasmic reticulum (ER) [30]. The viral NS proteins facilitate the formation of replication complexes by hijacking host cytoplasmic membranes. These compartments coordinate the replication and translation of the viral genome and help to shield viral components from host recognition. The NS proteins form a replication complex that generates negative-sense RNAs that function as templates for positive-sense genome RNA. Newly synthesized viral RNA is packaged, and the immature virion is transported through the host secretory pathway where it is further processed by host proteases to generate a mature virion that is released from the infected cell by exocytosis [6].

## 4. Host Innate Immune Response to *Flavivirus* Infection

The type I IFN signaling pathway is one of the first lines of defense against flavivirus infection of mammals. Type I IFNs are produced by mammalian cells in response to viral infection and play a pivotal role in counteracting viral pathogenesis [31]. Flavivirus-infected individuals have elevated levels of immune-related gene transcripts and serum IFN [32,33,34,35,36]. In mouse and cell models, similar elevations have been reported [37,38,39] and production has been shown to play a protective role [10,11,12,13]. Several proteins that function within this signaling pathway have been identified with direct and specific antiviral roles during flavivirus infection [37,38,40,41]. IFN-I has even been tested as a treatment for clinical flavivirus disease, but has not been successful [42,43]. This may be explained by the universal ability of flaviviruses to inhibit the Janus kinase-signal transducer and activator of transcription (JAK-STAT) pathway. As discussed below, the NS proteins are the primary actors in this suppression, and understanding the molecular mechanisms mediating this suppression would contribute to the development of effective antiviral therapies.

The innate immune response against flaviviruses is triggered by sensing the pathogen-associated molecular patterns (PAMPs) via the cytosolic and endosomal pattern recognition receptors (PRRs) retinoic acid-inducible gene-1 (RIG-I), melanoma differentiation-associated protein 5 (MDA5), Toll-like receptor (TLR) 7/8, or TLR3 [44,45,46]. PAMP sensing PRRs activate various kinases including inhibitor of nuclear factor kappa-B (NF-κB) kinase subunit epsilon (IKKε), tumor necrosis factor (TNF) receptor-associated factor (TRAF) family member-associated NF-κB (TANK)-binding kinase-1 (TBK-1), and TRAF3, which ultimately result in the phosphorylation of NF-κB and interferon regulatory factor 3 (IRF3) [45,47]. Activated NF-κB and IRF3 translocate to the nucleus to stimulate the production of type I IFNs.

The type I IFNs consist primarily of two secreted cytokines, IFN-α and IFN-ß. Upon secretion, IFN-α/ß bind to their cognate receptor IFNAR on infected and neighboring cells. IFNAR consists of two subunits (IFNAR1 and IFNAR2) whose intracellular domains are constitutively associated with the Janus kinases JAK1 and Tyk2. IFN binding to IFNAR activates JAK1 and Tyk2 to phosphorylate the latent cytoplasmic signal transducers of activation 1 and 2 (STAT1 and STAT2). Tyrosine phosphorylated STAT1 and STAT2 dimerize and then associate with a third protein, interferon regulatory factor 9 (IRF9). This trimeric complex, known as interferon-stimulated gene factor 3 (ISGF3), translocates into the nucleus where it binds to interferon stimulated response elements (ISRE) to drive transcription of over 300 interferon stimulated genes (ISGs) that directly or indirectly counter flavivirus infection. IRF9 binds to the ISRE, STAT2 contributes a potent transactivation domain, and STAT1 stabilizes the complex through additional DNA interactions [48,49].

The components of ISGF3 are constitutively expressed at a low level and reside in the cytoplasm in their latent forms. Upon IFN stimulation and tyrosine phosphorylation of STATs 1 and 2, ISGF3 rapidly assembles and directs a robust, transient antiviral response that includes the upregulation of ISGF3 components themselves. This response, however, additionally increases the expression of pro-apoptotic and anti-proliferative genes that can be damaging to the host cell, so downregulation of these genes occurs quickly after IFN stimulation. This response is mediated by several negative feedback mechanisms, such as the suppressor of cytokine signaling (SOCS) proteins, which are also induced by type I IFN [50]. In contrast, a subset of the antiviral ISGs upregulated by the initial IFN stimulation, including STAT1, STAT2, and IRF9, are sustained for several days, resulting in increased levels of the unphosphorylated forms of these proteins [51]. These proteins interact to form the trimeric unphosphorylated ISGF3 (U-ISGF3), which is responsible for the sustained ISG transcription, allowing for extended resistance to viral infection [52].

Recently, evidence has emerged for the existence of STAT1-independent complexes that can drive IFN-I-stimulated ISG expression [53]. For example, STAT2 can interact with IRF9 to form an “ISGF3-like” complex that activates ISRE-promoted genes in response to type I IFN [54]. This complex can direct a similar but prolonged ISGF3-like transcriptome in the absence of STAT1. However, some STAT2/IRF9-specific ISGF3-independent ISGs have been identified, including *CCL8* and *CX3CL1*. The promoter regions of these genes do not contain the classical ISRE sequences, suggesting that a DNA sequence distinct from ISRE may be involved in STAT2/IRF9-specific gene regulation. Additionally it has been demonstrated that this alternative pathway can mediate antiviral responses to several viruses including dengue, vesicular stomatitis, encephalomyocarditis, measles, Crimean-Congo hemorrhagic fever, and lymphocytic choriomeningitis viruses [55,56,57,58,59]. The cyclic guanosine monophosphate (GMP)–adenosine monophosphate (AMP) synthase (cGAS) is a cytosolic DNA sensor that directs the synthesis of cyclic GMP-AMP (cGAMP) upon binding to DNA. cGAMP activates the stimulator of the IFN gene (STING), which promotes type I IFN production via IRF3 activation [60]. It was recently demonstrated that the cGAS/STING pathway becomes activated during DENV infection, even in the absence of viral DNA intermediates [21]. DENV infection induces mitochondrial swelling, causing the release of mitochondrial DNA, which activates cGAS [61]. The involvement of the cGAS/STING pathway in other flavivirus infections has yet to be determined.

Although type I IFNs are produced by nearly all cells in the body and are essential for restricting viral replication, two additional IFN signaling pathways exist and have been shown to respond to viral infection. The type III IFNs (IFN-λ1-4) are the primary antiviral IFNs generated by epithelial cells and have similar functions and signaling pathways as IFN-I, but the cellular receptor is not ubiquitously expressed [62]. An antiviral effect for IFN-λ has been demonstrated for West Nile and Zika viruses. Mice deficient in the IFN-λ receptor exhibited increased blood–brain barrier (BBB) permeability after WNV infection [63], suggesting type III IFN signaling is involved in WNV neurotropism. Primary human trophoblast cells from human placenta were found to release type III IFN constitutively, conferring resistance to ZIKV infection [64]. The ability of ZIKV to be vertically transmitted from mother to fetus suggests the existence of a viral factor that may be able to overcome the type III IFN response in placental cells. The NS5 protein from ZIKV is a likely candidate as it was shown to inhibit the type III response in HEK293T cells [65].

While ZIKV NS5 was shown to suppress both the type I and III IFN responses, it was also able to activate type II IFN signaling [65]. The type II IFNs (IFN-γ) are generated mainly by immune cells and have some antiviral functions. ZIKV infection, however, is enhanced by type II IFN signaling, which generates proinflammatory cytokines that can facilitate viral spread and exacerbate Zika disease [66]. The concurrent NS5-mediated suppression of type I and III pathways and activation of the type II pathway was suggested to occur through increased homodimerization of STAT1, which upregulates gene expression at γ-activated sites (GAS). Because ZIKV NS5 induces the degradation of STAT2 (discussed below), which is required for the formation of transcription complexes involved in type I and III IFN signaling, the intracellular balance of STAT-containing complexes shifts to STAT1-STAT1 dimers, resulting in increased IFN-γ-induced gene expression. To date, ZIKV NS5 is the only viral protein known to concurrently suppress type I and III IFN pathways while activating type II [65].

## 5. *Flavivirus* Antagonism of Host Type I IFN Response

Just as hosts have evolved multiple mechanisms for inhibiting viral infection, viral proteins have gained the ability to antagonize the host IFN response over time. One mechanism by which the type I IFN response is passively avoided by flaviviruses is evasion of the host PRRs, described above. Flaviviruses encode their own methyltransferase that caps the RNA genome to mimic the RNAs present in the host cell. The cap structure hides the viral genome from members of the interferon-induced tetratricopeptide repeats (IFIT) protein family, which binds to and sequesters viral RNA, and prevents recognition by the RIG-I [67]. Flaviviruses also shield their genome from host sensing by enclosing their replication complex (RC) in membranes on the ER surface, a mechanism that has been observed in the early stages of DENV, WNV, and tick-borne encephalitis virus (TBEV) infections [68,69]. However, late in the infection, newly synthesized viral RNA is abundant, and the RC loses some integrity, which may lead to the release of RNA intermediates that could activate RIG-I or MDA5 signaling [70].

Flaviviruses have also been demonstrated to actively abrogate the activity of protein functioning within the type I IFN signaling pathway. One common mechanism is interference with post-translational modifications of these proteins. For example, the DENV, WNV, and YFV NS4B inhibit STAT1 phosphorylation [71,72]. WNV NS4B is additionally implicated in preventing the phosphorylation of JAK1 and Tyk2 [27,28], and NS2A, NS2B, NS3, NS4A, and NS4B from Kunjin virus (KUN), a close relative to WNV, are all implicated in JAK-STAT inhibition [73]. This mechanism is not always mediated by NS proteins, however. Flaviviruses produce small RNAs called subgenomic flavivirus RNAs (sfRNAs) that are generated by the incomplete degradation of the viral genome by the host endonuclease XrnI [74]. In DENV, this sfRNA binds to TRIM25, which normally interacts with RIG-I to promote its ubiquitination and interaction with mitochondrial antiviral signaling protein (MAVS) [75]. The DENV sfRNA prevents the deubiquitination of TRIM25, an essential upstream activator for RIG-I activation [76].

Another mechanism of active interference with the type I IFN system is competitive binding. DENV and WNV NS3 proteins compete with RIG-I for 14-3-3ε binding, a chaperone responsible for trafficking RNA-bound RIG-I to the mitochondrial membrane [77]. Additionally, the DENV NS4A protein sequesters MAVS, preventing RIG-I-MAVS interaction, IRF3 activation, and IFN-I production [78].

Finally, many flavivirus NS proteins have evolved mechanisms to degrade host immune proteins, or to induce the degradation of these proteins by hijacking the host proteasome system. For example, the DENV NS2B/NS3 viral protease suppresses the DNA sensing pathway and RIG-I sensing by cleaving STING, resulting in reduced type I IFN production [21]. Additionally, NS2B by itself promotes the autophagy-lysosome-dependent degradation of cGAS [21].

## 6. NS5 Structure and Function

NS5 is the most conserved flavivirus protein, with less than 45% amino acid difference reported among the vector-borne flaviviruses [3]. The N-terminus encodes the viral methyltransferase (MTase), while the C-terminus encodes the RNA-dependent RNA polymerase (RdRp) [79]. The RdRp generates positive- and negative-sense RNAs from the RNA genome de novo [80,81], and the replication process is thought to involve three different conformational states: pre-initiation, initiation, and elongation [82,83]. The MTase domain caps the RNA genome via a two-step reaction and also serves as the guanylyltransferase [29].

To date, structures of full-length NS5 from JEV [84], DENV3 [85], and ZIKV [79,86,87] have been reported. The ZIKV and JEV NS5 conformations exhibit a high degree of similarity, suggesting structural conservation among the flaviviruses [79]. In contrast, the domain orientations of ZIKV and DENV NS5s differ significantly, although the residues at the domain interface are highly conserved among ZIKV, DENV, and JEV [79]. These observations support an earlier observation that the flavivirus NS5 exhibits a high degree of flexibility in solution and can adopt a compact or extended conformation [88], which may impact on the diverse functions of NS5. Indeed, mutagenesis of the conserved interface residues of the DENV3 NS5 caused enhanced RdRp activity, but inhibited viral infectivity [85]. Furthermore, recent structural evidence suggests that the two subdomains cooperate in the execution of the sequential replication and capping functions, although the mechanism remains unknown [84,89]. Taken together, these reports suggest that the flexibility and distinct conformations of the flavivirus NS5 may be linked to the various steps involved in RNA capping and replication. More experiments are needed to clarify the potential role of the conformational changes in regulating NS5 activities.

Several post-translational modifications of flavivirus NS5s have also been documented with potential regulatory roles. Serine/threonine phosphorylation appears to be conserved throughout the *Flaviviridae* family [90,91], but the function of this modification and the identity of the host kinases involved are largely unknown. The DENV NS5 protein is phosphorylated by both mammalian and mosquito protein kinase G at a conserved Thr449 in the RdRp domain [92,93]. The differential phosphorylation of DENV NS5 was shown to affect its interaction with the viral helicase, NS3, which is required for genome replication [94]. The WNV NS5 MTase domain is also phosphorylated by, and interacts with, protein kinase G, and abolishing this interaction inhibits viral replication [95]. SUMOylation of the DENV NS5 was shown to be important for DENV replication [96]. In addition, glutathionylation of the DENV and ZIKV NS5s has been reported [97].

Replication of the flavivirus genome occurs exclusively in the cytoplasm [69,98]. However, a significant portion of the NS5 protein is observed within the nucleus during YFV [99], JEV [100], ZIKV [3], WNV [101], and DENV [102] infections. Inhibiting the nuclear localization of DENV and WNV NS5 significantly decreases viral titers [101,103]. Inversely, inhibiting the nuclear export of DENV NS5 decreased the induction of IL-8, which plays a role in induction of inflammation [104], suggesting nuclear localization of NS5 may be important for immune modulation [105]. This observation is further supported by a study showing that interaction of NS5 with host spliceosome components leads to changes in mRNA isoform abundance of antiviral factors [106]. There are differences, however, in the level of NS5 nuclear localization among the different DENV serotypes. NS5 from serotypes 2 and 3 accumulate in the nucleus, while DENV1 and 4 NS5 reside in the cytoplasm [107]. For serotype 4, cytoplasmic localization is likely due to the lack of a functional nuclear localization signal (NLS) [107]. Levels of IL-8 did not change with the different serotypes, and the function of the differential localization is still unknown [107].

## 7. IFN Suppressor Function of *Flavivirus* NS5 Protein

While multiple proteins capable of IFN antagonism have been described for the major disease-causing flaviviruses, NS5 is the most potent and direct antagonist [3]. This is significant because NS5-mediated IFN antagonism is required for counteraction of IFN in cell culture [108] and for virulence in mouse models [109]. Remarkably, even though this protein utilizes similar MTase and RdRp mechanisms no matter the flavivirus species to replicate and cap their RNA genomes, the mechanism of NS5-mediated IFN suppression diverges within the genus. The evolution and divergence of this role for NS5 may have been facilitated by the strategy of expression of flaviviral proteins from a single open reading frame. This results in excess expression of NS5, while only small amounts are needed for RdRp and MTase functions. Due to the lack of high-resolution structures for the complexes of various NS5s and their interacting host partners, how NS5 is able to retain its roles as MTase and RdRp while evolving divergent IFN suppression mechanisms remains elusive.

NS5 frequently employs multiple strategies to suppress the JAK-STAT signaling pathway not only among different species of *Flavivirus,* but also within the same species. For example, the ZIKV NS5 protein has been shown to both inhibit the phosphorylation of STAT1 and induce the degradation of STAT2. The JEV, DENV, and WNV NS5 proteins have similarly been shown to antagonize this pathway at multiple steps. STAT2 is a common target for NS5-mediated IFN suppression, and at least two flavivirus NS5 proteins have been demonstrated to target this protein for degradation. The mechanisms by which the DENV and ZIKV NS5s degrade STAT2 diverge, however, and the molecular details of these mechanisms are still being elucidated.

### 7.1. Dengue Virus

DENV NS5 binds to human STAT2 and inhibits its phosphorylation, resulting in reduced ISG transcription. The mechanism of this inhibition has not been elucidated, but the IFN suppression activity was mapped to the RdRp domain [110]. Expression of NS5 also reduces IFN-α-, but not IFN-γ-, mediated STAT1 phosphorylation, although NS5 does not directly interact with STAT1 [110].

Ashour et al. demonstrated STAT2 degradation as an additional mechanism of DENV NS5-mediated IFN antagonism [111,112]. However, while this study also found that binding of NS5 to STAT2 is sufficient to prevent IFN signaling, STAT2 degradation is detected only when the N-terminus of NS5 is proteolytically processed, as it would be in the context of viral infection. NS5 is separated from NS4B during polyprotein processing by the viral NS2B/NS3 protease, and co-expression of NS5 with NS2B/NS3 induces STAT2 degradation. Replacement of the viral cleavage site at the N-terminus of NS5 with a host protease cleavage site, however, also allows for the efficient degradation of STAT2, suggesting the cleavage does not need to be mediated by the viral protease. Additionally, the identity of the N-terminal residue of NS5 does not appear to be important to this processing event, as replacing the glycine residue at position 1 of NS5 with methionine resulted in efficient STAT2 degradation [112]. This study also demonstrated that NS5-mediated STAT2 degradation is dependent on the ubiquitin-proteasome pathway, implying the involvement of a host E3 ligase. In a follow-up study, the García-Sastre group identified this protein as UBR box N-recognin-4 (UBR4), which is part of the N-recognin family [113]. Members of this family target proteins that undergo conformational changes to expose a destabilizing N-terminal residue for degradation, a mechanism for the N-end rule pathway [114]. UBR4 binds to the first five amino acids of NS5; deleting the first ten amino acids of NS5 eliminates its ability to induce STAT2 degradation [112,113]. The binding domain for STAT2, however, was mapped to residues 202–306 [112], suggesting that the DENV NS5 central and N terminal regions together serve as a bridge between STAT2 and UBR4. In this scenario, it is possible that NS5 and STAT2 are both targeted by UBR4 and similarly degraded [7]. Experimental evidence, however, is still needed for verification of the role of the N-end rule pathway in DENV NS5-mediated STAT2 degradation.

### 7.2. Zika Virus

Similar to DENV NS5, ZIKV NS5 binds to human STAT2, triggering its degradation. However, unlike what was observed for DENV NS5, ZIKV NS5 does not need to undergo proteolytic processing for depletion of STAT2 [3]. Additionally, it was demonstrated that the first ten amino acids of NS5 are dispensable for depletion of STAT2, suggesting that the N-end rule does not apply to ZIKV NS5 [115]. STAT2 is ubiquitinated prior to degradation, and proteasome inhibitors rescue STAT2 protein levels in the presence of NS5 [65]. However, unlike that occurs for DENV NS5, UBR4 is not involved in mediating STAT2 degradation [3]. This suggests that ZIKV NS5-mediated STAT2 degradation utilizes the host ubiquitin proteasome system and the participating host E3 ligase has yet to be identified.

Chaudhary et al. demonstrated an additional consequence of ZIVK NS5-mediated STAT2 degradation, aside from decreased ISG induction. In an uninfected cell, unphosphorylated STAT2 can bind to both unphosphorylated and phosphorylated STAT1 to prevent translocation of STAT1 and activation of the type II IFN response [116]. In ZIKV-infected and NS5-transfected cells, however, an increase in STAT1 homodimerization is observed, concurrent with an increase in type II IFN and a decrease in type I IFN signaling. In this model, the degradation of STAT2 frees up STAT1 proteins to homodimerize and translocate to the nucleus to selectively activate ISGs controlled by gamma activated sites (GAS) [65].

### 7.3. Yellow Fever Virus

The YFV NS5 also targets STAT2 as part of its IFN-I suppression mechanism. YFV NS5 binds to STAT2, but this interaction is uniquely dependent on host cell stimulation with type I or III IFNs. This stimulation induces several intracellular events required for NS5 association with STAT2. First, as in an uninfected cell, stimulation with IFN induces the phosphorylation and heterodimerization of STAT1 and STAT2. STAT2 does not need to be phosphorylated for NS5 interaction. Instead, the association of STAT1 and STAT2 induces a conformational change within STAT2 that allows for NS5 binding. Second, IFN stimulation promotes the ubiquitination of YFV NS5 by TRIM23; non-ubiquitinated NS5 cannot interact with STAT2. Unlike the DENV and ZIKV NS5s, YFV NS5 does not induce the degradation of STAT2, and is able to bind STAT2 both in the cytoplasm and in the nucleus. YFV NS5 blocks IFN production either by directly interfering with ISGF3 binding to ISRE promoter elements in the nucleus, or by preventing IRF9 association with STAT1/2 heterodimers in the cytoplasm. A domain mapping study identified the first ten amino acids of the YFV NS5 as essential for both STAT2 interaction and IFN-I inhibition, consistent with the requirement of TRIM23 ubiquitinating K6 of NS5 [117].

## 8. Other NS5 Interactions

### 8.1. Spondweni Virus

Spondweni virus (SPOV) is the closest known relative of ZIKV [118], and their NS5 proteins share 77% amino acid identity [3]. While SPOV NS5 does not directly interact with STAT2 as the ZIKV NS5 does, it is an inhibitor of JAK-STAT signaling, as SPOV NS5-transfected cells inhibit ISRE-dependent gene expression [3]. The STATs are also phosphorylated and translocated to the nucleus upon IFN stimulation. SPOV NS5 is localized primarily to the nucleus of the cell, so it is possible the mechanism of ISG suppression occurs inside the nucleus [3].

### 8.2. Japanese Encephalitis Virus

The JEV NS5 protein alone can inhibit Tyk2 and STAT1 phosphorylation via protein tyrosine phosphatase (PTP) activity, as PTP inhibitors rescue phosphorylation, but specific NS5 interactions with innate immune proteins have not yet been implicated in this suppression mechanism [119]. The region of NS5 required for suppression of STAT1 activation was mapped to the N terminus [119]. JEV NS5 has also been shown to inhibit the nuclear translocation of IRF3 and NF-κB by competitively interacting with importin-α4 and importin-α3 [120]. These interactions are mediated by the NLS of JEV NS5 and mutagenesis of key residues in this region restored ISG expression [120].

### 8.3. Tick-Borne Encephalitis, Langat, and West Nile Viruses

Langat virus (LGTV) is a member of the tick-borne encephalitis virus (TBEV) serogroup. Both LGTV and TBEV NS5s suppress phosphorylation of STAT1, STAT2, Tyk2, and JAK1 [121]. The region of LGTV NS5 responsible for this suppression mechanism was mapped to the RdRp domain of NS5 [122]. The minimal linear sequence required was mapped to residues 355–735 which overlap with the finger region and the eight conserved RdRp motifs [122]. The specific amino acids required lay within two noncontiguous stretches of amino acids: 374–380 within the finger domain and 624–647 within the palm domain [122]. When modeled on the crystal structure of the WNV RdRp, these two amino acid stretches are adjacent to one another, suggesting cooperative action [122].

It was later determined that LGTV and TBEV NS5s also interact with prolidase (PEPD), a host protein that is required for IFNAR1 maturation [109]. This interaction was mapped to the same region as that required for LGTV NS5-mediated STAT1 phosphorylation inhibition [121]. STAT1, STAT2, Tyk2, and JAK1 phosphorylation occur downstream of IFNAR1, so this NS5 interaction elucidated the mechanism of NS5-mediated IFN suppression in addition to the mechanism of IFNAR1 downregulation. LGTV and TBEV NS5s are also known to interact with IFNAR2 and IFNGR1, but the function of these interactions is still unknown [121].

TBEV NS5 has also been shown to interact with the mammalian membrane protein Scribble which has been implicated in T cell activation [123]. The interaction was mapped to the MTase domain of TBEV NS5 and the PSD-95, Discs-large, ZO-1 domain 4 (PDZ4) of Scribble [123]. In IFN-stimulated cells depleted for Scribble, phosphorylation and nuclear localization of STAT1 was restored [123].

WNV NS5, like LGTV and TBEV, was also shown to interact with PEPD to downregulate IFNAR expression [110]. This interaction may be the mechanism by which WNV NS5 inhibits STAT1 and STAT2 phosphorylation, but further investigation is required for this conclusion [3,108].

## 9. Summary

Effective inhibition of type I IFN production is necessary for flaviviruses to establish infection in mammalian hosts. The viral non-structural proteins have evolved to be multi-functional, encoding diverse IFN suppression mechanisms in addition to their essential roles in the viral life cycle. NS5 is one of the most important IFN-I antagonists. Three of the most pervasive disease-causing flaviviruses—YFV, ZIKV, and DENV—inhibit human STAT2 through NS5-hSTAT2 interaction. Detailed mechanistic understanding of these interactions provides at least two opportunities for translational research. First, recombinant viruses that incorporate loss-of-function mutations in NS5 are attractive candidates for live-attenuated vaccine strains. Second, antivirals that target the NS5-hSTAT2 interaction would inhibit an early step common to these flaviviruses, despite divergent downstream mechanisms employed by the NS5s. These applications require deeper mechanistic understandings of the interaction between NS5 and hSTAT2.
